# The Guyana Diabetes and Foot Care Project: Improved Diabetic Foot Evaluation Reduces Amputation Rates by Two-Thirds in a Lower Middle Income Country

**DOI:** 10.1155/2015/920124

**Published:** 2015-05-19

**Authors:** Julia Lowe, R. Gary Sibbald, Nashwah Y. Taha, Gerald Lebovic, Madan Rambaran, Carlos Martin, Indira Bhoj, Brian Ostrow

**Affiliations:** ^1^Division of Endocrinology, Department of Medicine, University of Toronto, Toronto, ON, Canada M5S 1A8; ^2^Institute of Health Science Education, University of Guyana, Georgetown, Guyana; ^3^Diabetic Foot Centre, Georgetown Public Hospital Corporation, Georgetown, Guyana; ^4^Department of Surgery, University of Toronto, Toronto, ON, Canada M5T 1P5

## Abstract

*Background*. Type 2 diabetes is the fourth leading cause of death in Guyana, South America. A complex, interprofessional, quality improvement intervention to improve foot and diabetes care was rolled out in two phases. *Methods & Findings*. Phase 1: Establishment of an Interprofessional Diabetic Foot Center (DFC) of Excellence to improve foot care and reduce diabetes-related amputations at the national referral hospital. Phase 2: Regionalization to cover 90% of the Guyanese population and expansion to include improved management of diabetes and hypertension. Fourteen key opinion leaders were educated and 340 health care professionals from 97 facilities trained. Eight centers for the evaluation and treatment of foot ulcers were established and 7567 people with diabetes evaluated. 3452 participants had foot screening and 48% were deemed high risk; 10% of these had undocumented foot ulcers. There was a 68% reduction in rate of major amputations (*P* < 0.0001); below knee amputations were decreased by 80%, while above knee amputations were unchanged. An increased association of diabetes with women (F/M = 2.09) and increased risk of major amputation in men [odds ratio 2.16 (95% CI 1.83, 2.56)] were documented. *Conclusions*. This intervention improved foot care with reduction in major amputations sustained over 5 years.

## 1. Introduction

While substantial research has demonstrated the potential for preventing the adverse outcomes of type 2 diabetes [1], the increase in the number of people with diabetes (PWD) has outpaced the response of health systems [2, 3]. This incongruity is particularly marked in low and middle income countries (LMIC) where 80% of deaths from diabetes occur [4].

The estimated adult prevalence of diabetes in Guyana was 15.5% in 2011 [5] and in 2008 diabetes was the fourth leading cause of death [6]. Prior to 2008, diabetic foot complications were the most common admitting diagnosis to the surgical ward of the national referral and teaching hospital, Georgetown Public Hospital Corporation (GPHC) [7], with 42% having a lower extremity amputation (LEA) [8]. Scoping visits by Canadian expert team members participating in a recently introduced surgery residency program [9] confirmed that diabetic foot complications were the most common reason for admission to the surgical units of GPHC. Care was uncoordinated, with lack of a systems approach (no screening for high risk feet/ulcers, practice in silos, overaggressive debridement without adequate vascular assessment, no plantar pressure redistribution, and narrow spectrum or missed antimicrobial doses). The high burden of disease (30% inpatient population), prolonged stay, and frequent readmissions with poor patient outcomes resulted in staff demoralization. Surgical debridement took place either on poorly lit and unsanitary wards or had to compete with other surgical emergencies in the operating rooms. There was inappropriate reliance on major amputations instead of limb salvage. Local health care leaders were keen to address this problem and entered into a partnership with Canadian surgeons and wound/foot care experts to develop the Guyana Diabetes and Foot Care Project (GDFP) 2008–2013.

The Phase 1 goal was to create health system changes in evaluation and management to improve foot care in PWD and reduce diabetes-related LEA at GPHC, while Phase 2 expanded this to 6 administrative regions, comprising 90% of the population, and added training in the management of diabetes and hypertension.

## 2. Materials and Methods

Clinical activities resided within the Guyanese public health system and staff and resource costs were paid by Ministry of Health (MoH). Multilevel knowledge to action (K2A) cycles was utilized to identify the challenges facing the Guyanese health care system and develop the intervention. Both process and clinical outcomes were monitored. Participants were all persons with type 2 diabetes presenting to the GPHC or to regional facilities with personnel trained in the project, and the care provided to them was based on the decision of these personnel and patient wishes. Patient data was entered into a ministry approved database and identifiable personal information, apart from sex and age, was withheld from the authors. Since this was a quality improvement (QI) project, run under the auspices of the Ministry of Health of Guyana in public health facilities, approval of an ethics committee was not required. Project oversight and coordination was provided by steering committees, meeting regularly, with both Canadian and Guyanese members, including Ministry of Health officials.

### 2.1. Project Model

The key interventions are detailed elsewhere [10]. In brief, the intervention comprised development of a key opinion leader (KOL) team, this following a train the trainer model. The KOLs were trained using well established Canadian training programs: the International Interprofessional Wound Care Course (IIWCC) [11] and the International Diabetes Federation approved Michener Institute Diabetes Educator course. Health systems change was facilitated through networking with key stakeholders to establish foot care centres and embed practice change. Key opinion leaders (KOLs) attended these training programs; then trained primary health care workers through iterative 3-day workshops on basic foot and wound care using the screening tool and referral criteria. All trainings were interprofessional with doctors, nurses, medex (doctor equivalents), and rehabilitation specialists learning together. The Canadian training programs were supplemented by on-site skills training and reflective practice to develop local expertise as well as supported by continued mentoring from Canadian experts.

### 2.2. Diabetic Foot Evaluation and Management

Given the limited local resources, it was important to allocate the available resources effectively, and this was facilitated by using clinical screening tools to recognize loss of protective sensation, and identification of the patient at high risk of ulceration or amputation. The simplified 60-second screening tool was developed [12]. The highest risk individuals were then referred to the national Diabetic Foot Center (DFC) at the Georgetown Public Hospital Corporation (GPHC) [13] for more intensive surveillance, education on foot care foot wear and smoking cessation, debridement of callus linked to the use of protective footwear and orthotic devices, improved glycemic control, and the treatment of foot ulcers/complicating infections [14]. Absence and cost of wound care products used for diabetic foot ulcer care in high income countries led to adaptation of more cost effective wound care practices, and commercially available (Darco) wound care sandals were prescribed at a fraction of the price. In the absence of any foot specialists, principles of plantar pressure redistribution (PPR) therapy were taught to rehabilitation assistants, orthotic, prosthetic, and cast room technicians. In Phase 2, the previously listed methods were applied to build capacity across the country, and the foot care program was expanded to 6 Guyanese administrative regions. A 3-day training program on diabetes and hypertension management was added with these components introduced throughout the project regions and HbA1c testing introduced into the public system. A project database was designed to capture the more complex project outcomes with clerks appointed and trained in data entry at each center.

### 2.3. Targeted Outcomes

Targeted process outcomes were the establishment of a National Centre of Excellence in foot care at GPHC and 7 regional foot care centers, project tools accepted and used by the MoH, measurement of HbA1c and blood pressure for people with diabetes, identification of the high risk foot using the simplified 60-second screening tool, and appropriate referral to regional or national DFCs. Targeted clinical outcomes included reduction in major LEA at GPHC and measurement of the proportion of PWD with HbA1c <9% (75 mmol/mol) and BP <160/95. Diabetic foot admissions at GPHC were determined using admission books on surgical wards and amputations from operating room records. Audits of the admissions book on surgical floors were undertaken to identify patients with diabetic foot complications from 2006 to 2010 because a review of chart coding based records found it to be inaccurate.

### 2.4. Statistical Analysis

**Figure 1:**
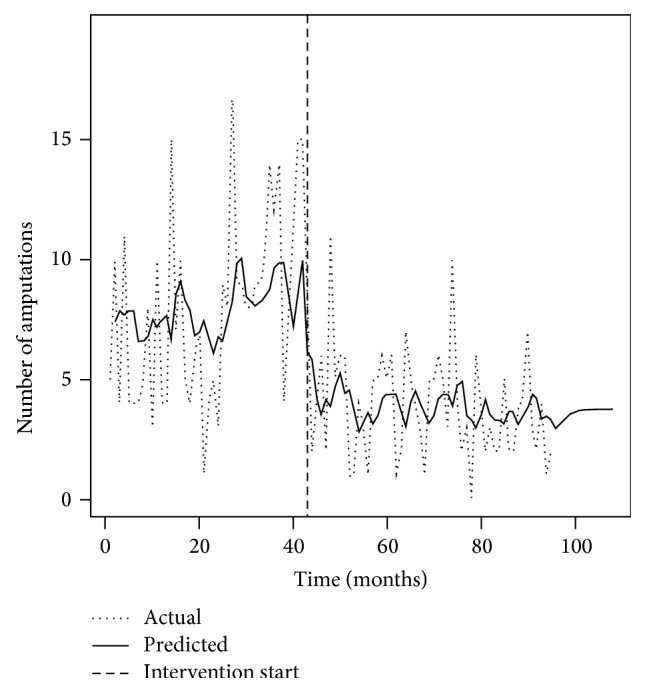
Time series analysis of diabetes-related major amputations at Georgetown Public Hospital Corporation 2005–2012.

Continuous variables were summarized using means (SD) and median (IQR) and tested using two sample *t*-tests and paired *t*-tests as appropriate. Although the *t*-test is robust to nonnormality, since some data was mildly skewed, we verified the results using a nonparametric Wilcoxon test and found similar results. Categorical data was reported using frequency and percent and tested using the Chi-Square statistic. Odds ratios were also examined for comparisons between groups and the Breslow-Day statistic was used to test for the homogeneity of odds ratios. Time series analysis was employed to examine the effect of the intervention on the number of amputations after adjusting for autocorrelation and is presented in Figure 1. Autocorrelation and partial autocorrelation plots indicated a good fit. The augmented Dickey-Fuller test and Ljung-Box test indicated good fitting and no concern due to stationary or white noise [15–17].

## 3. Results and Discussion

### 3.1. Educational Outcomes

Key opinion leader (KOL) team: A total of 16 trainees (7 doctors, 1 medex, 4 nurses, 3 rehabilitation specialists, and 1 diabetic foot care worker) participated in the International Interprofessional Wound Care Course in 5 cohorts; 14 completed the course and 10 are currently working in the KOL team. The KOL team then trained a total of 340 other Guyanese health care professionals (F/M = 1.8) (Phase 1: 65 HCP in 4 workshops; Phase 2: 275 HCP in 18 workshops). These professionals staff 8 DFCs and 89 health facilities providing chronic disease care.

### 3.2. Clinical Outcomes

#### 3.2.1. Foot Screening

The simplified 60-second screening tool was developed in Guyana [12] and implemented to screen 3452 persons and 643 completed a follow-up screen. 48% had at least one abnormality and were classified as high risk. A reliability study confirmed the utility of this tool [18], which was adopted by the MoH to be used throughout Guyana.

#### 3.2.2. Patient Database

From July 2010 to March 2013, 7567 PWD were assessed with F/M = 2.09 [19]. As of March 2010, there were a cumulative 6075 patient visits to the GPHC foot center, an average of 13.6/day. As of March 2013, 1186 patients (F/M = 1.60) with foot ulcers have been treated at regional DFCs; there have been over 20,776 visits for dressing care. HbA1c testing was successfully introduced to the public system and a tool to ensure appropriate use of this limited resource was implemented. Since April 2010, 4062 PWD have had HbA1c testing of whom 62% had an HbA1c <9% (75 mmol/mol). The average HbA1c was 8.56% (SD ± 2.85) {70 mmol/mol; SD ± 28 mmol/mol} with women having significantly higher values than men 8.66 (SD ± 2.89) {71 mmol/mol; SD ± 31.6 mmol/mol} versus 8.31 (SD ± 2.77) {67 mmol/mol; SD ± 30.3 mmol/mol}; *P* = 0.0001. Mean HbA1c was 13% higher in patients with foot complications with 44% having HbA1c over 9% (75 mmol/mol). The average blood pressure in 814 PWD was 134 mmHg systolic and 82 mmHg diastolic. 16% of patients had blood pressure greater than either 160 systolic or 95 diastolic. 30% had blood pressure greater than either 140 systolic or 90 diastolic. 649 persons (80%) were on treatment for hypertension. There was not enough power to detect a change in BP or HbA1c over time as too few subjects had recurrent measures. Change in these outcomes is likely to be incremental rather than sudden.

#### 3.2.3. Diabetes-Related Major Lower Extremity Amputations at GPHC

In the 42 months before the DFC opened, the mean monthly number of amputations was 7.95 (SD ± 4.05) and this fell significantly to 3.89 (SD ± 2.30) in the 54 months after the DFC opened through to December 2012 (*P* < 0.0001). This represents a 51% decrease and translates to a saving of 219 limbs from July 2008 to December 2012. The time series analysis (Figure 1) demonstrated a significant decrease in the number of amputations (4.32/month (95% CI 2.40, 6.24); *P* < 0.0001) coincident with the commencement of the project. The opening of the DFC overlapped an ongoing postgraduate surgical training programme [9]. While an apparent rise in major amputation numbers during months 24–41 may have been associated with this increase in surgical capacity, this did not preclude the observed reduction in monthly amputations subsequent to DFC operation.

Of even greater significance is the marked reduction in proportion of inpatients with diabetic foot complications subjected to major amputation (Table 1) despite a doubling of the rate of diabetic foot admissions at GPHC. The average monthly admissions rose from 21.2 before the DFC opened to 42 in the first 22 months of operation. The proportion of inpatients subjected to a major amputation during this period fell from 41.4% to 11.9% (*P* < 0.0001). A Poisson regression model was used to examine the rate of amputations adjusted for patient volume and after intervention the risk of having an amputation decreased by 68.6% (95% CI 53.9%, 78.5%) as compared to before intervention.

The number of specific types of major amputations, their means, and medians are shown in Table 2. The sums are less than the total number of amputations reported because the type was not specified in some cases. There was no significant difference (*P* = 0.07) in the mean number of above knee amputations (AKA) after intervention as compared to before, while there was a significant difference in below knee amputations (BKA) (*P* < 0.0001).

The changes in the frequency of AKAs and BKAs before and after the intervention give an indication of the limitations of this kind of project focused on primary care. While BKAs showed an 80% and significant reduction after the DFC was opened, AKAs showed no change. Currently there is no vascular surgical capacity in Guyana to treat vascular insufficiency, a common comorbidity in diabetic foot complications. We suggest that patients with both diabetic foot complications and uncorrected vascular insufficiency are more likely to require AKAs. This service gap could explain the lack of decline in AKAs after intervention. It would also speak to the need for developing a vascular surgical capacity in resource-constrained settings, if limb salvage in the diabetic foot is to be optimized. Figure 1 illustrates a plateau effect on amputations after intervention and it may be that this is the best that can be achieved without further resources (e.g., vascular surgery and renal dialysis).

### 3.3. Sex-Based Differences in Type 2 Diabetes and Amputations

We have already reported on the divergence from global averages of the sex ratios of type 2 diabetes in Guyana and have estimated that the odds ratio for women compared to men is 2.486 (95% CI 2.442, 2.531, *P* < 0.0001) [19]. The sex-based risks for diabetes-related amputations in Guyana are reversed. There were 544 major amputations (278 in women and 266 in men) over 8 years with F/M of 1.05. Since few regional hospitals in Guyana have surgical capacity and most diabetic foot problems are referred to GPHC, virtually all diabetes-related LEAs in Guyana are being performed at that hospital. To calculate the sex-based relative risks we assumed that any amputations outside GPHC follow the same distribution for sex and type and that the number of persons with diabetes remained constant over the 2005–2012 periods. Since the estimated prevalence of diabetes in women is twice that in men, the odds ratio of amputations for males as compared to females during the entire study period is 2.16 (95% CI 1.83, 2.56; *P* < 0.0001). The reasons for the increased risk of amputations in men are unknown but may be related to an increased risk of ulceration due to social factors (occupational hazards, smoking) or failure to seek medical attention. The odds ratios for amputations in men compared to women increased from 1.86 (95% CI 1.50, 2.31) before the intervention to 2.73 (95% CI 2.08, 3.58) after the intervention (*P* = 0.015). We tested whether AKA and BKA amputation rates differed between males and females before versus after the intervention and found that the odds ratios did not change before versus after intervention (OR = 0.9959 {95% CI 0.6576, 1.4682}).

### 3.4. System Change

The MoH embraced the model, which is described in detail elsewhere [10], and approved in the new* MOH Strategic Plan 2013–2020: Integrated Prevention and Control of Non Communicable Disease in Guyana* [20]. Despite the many challenges facing the MoH, a significant change in the approach to evaluation of the diabetic foot and diabetes management occurred.

## 4. Conclusions

We demonstrated that it is possible to introduce the best practice methods to evaluate for the high risk foot in people with diabetes and achieve sustained improvements in evaluation and care of foot ulcers. After the project began GPHC achieved a marked and sustained reduction both in major amputation numbers and in the proportion of inpatients with diabetic foot complications requiring major amputation. That this reduction occurred almost immediately after project commencement suggests that surgeons embraced the importance of maintaining limb integrity. Change was likely sustained by provision of new alternate methods and dedicated clinic spaces for treatment based on context specific practice guidelines. Vascular surgery capacity is essential to maximize limb salvage.

Translating clinical guidelines and QI principles into practice, in both the developed and developing world is challenging. In low and middle income countries (LMIC) the challenge is to deploy interventions that are cost saving or cost effective. This requires empirical research in a variety of contexts. Our project contributes to this research. One of our next steps is to investigate the transferability of our model to another limited resource setting.

## Figures and Tables

**Table 1:** Diabetic foot (DF) admissions and amputation rates at Georgetown Public Hospital Corporation.

Variable	Before DFC (30 months)	After DFC (22 months)	Analysis
DF admissions (ward records)	633	924	
Number of amputations	262	110	
Average monthly proportion DF patient with major amputation	41.4%	11.9%	*P* < 0.0001

**Table 2:** Major amputations by type at Georgetown Public Hospital Corporation.

Variable	Before intervention	After intervention	Test statistic	*P* value
Time in months	42	48		
*N* above knee amputations	124	113^*^		
Mean (SD)	2.95 (2.44)	2.13 (1.81)	−1.82 (t)	0.07
Median (IQR)	2 (1–4)	2 (1–3)	1.47 (Z)	0.14
*N* below knee amputations	166	41^*^		
Mean (SD)	3.95 (2.64)	0.77 (1.05)	−7.35 (t)	<0.0001
Median (IQR)	3 (2–5)	0 (0-1)	6.82 (Z)	<0.0001

^*^Represents total with available dates. One AKA and 3 BKAs were not dated.

## Abbreviations

AKA: Above knee amputation
BKA: Below knee amputation
BP: Blood pressure
CI: Confidence interval
CIDA: Canadian International Development Agency
CME: Continuous medication education
DFC: Diabetes and foot center
F/M: Female to male ratio
GDFP: Guyana Diabetes and Foot Care Project
GPHC: Georgetown Public Hospital Corporation
HbA1c: Glycosylated hemoglobin
HCP: Health care professional
IDF: International Diabetes Federation
IIWCC: International Interprofessional Wound Care Course
K2A: Knowledge to action
KOL: Key opinion leader
LEA: Lower extremity amputation
LMIC: Low and middle income countries
MoH: Ministry of Health
PPR: Plantar pressure redistribution
PWD: Person with diabetes
QI: Quality improvement
SD: Standard deviation.
